# The first complete chloroplast genome of *Quercus coccinea* (Scarlet Oak) and its phylogenetic position within Fagaceae

**DOI:** 10.1080/23802359.2019.1677189

**Published:** 2019-10-18

**Authors:** Xuemei Yang, Yanlei Yin, Lijuan Feng, Haixia Tang, Fei Wang

**Affiliations:** Shandong Institute of Pomology, Shandong Academy of Agricultural Sciences, Taian, Shandong, People’s Republic of China

**Keywords:** *Quercus coccinea*, chloroplast genome, illumina sequencing

## Abstract

*Quercus coccinea* is a native to North America, which grows well in acidic soil and resists cold to extreme low temperature –40 °C. The chloroplast (cp) genome of *Q. coccinea*, sequenced based on next-generation platform (NEOSAT), is 161,298 bp in size. The cp genome encodes 133 genes, including 88 protein-coding genes (PCGs), 8 rRNA genes, and 37 tRNA genes. Phylogenetic relationship analysis based on complete cp genome sequences exhibited that both of *Q. coccinea* and *Q. rubra* were phylogenetically closer to *Q. aliena* var. *Acutiserrata*.

*Quercus coccinea*, known as the Scarlet Oak, is a native to North America. It is a deciduous tree of the family Fagaceae and reaches a height of 22 m and a spread of 12–15 m (Yuan and Tao 2010). The leaves, more deeply lobed than *Q. rubra*, turn scarlet in fall. It flowers in May, with fruit ripening in October. It grows well in acidic soil and resists cold to extreme low temperature –40 °C (Yuan and Tao 2010). It is difficult to transplant successfully. *Quercus coccinea* is well suited for planting as street tree because of its rounded, spreading canopy and beautiful fall leaves (Yuan and Tao 2010). It is difficult to distinguish and identify the oak trees based on morphological characteristics. Thus the genomic and genetic information is urgently needed to facilitate the genetic marker, molecular evolution and systematics research of *Q. coccinea*.

The voucher specimen (accession no. TA_2057_XHL_Taishan) was harvested from a mature *Q. coccinea* tree on the campus grounds of Shandong Institute of Pomology (SDIP) in Taian, Shandong, China (36.20°N, 117.12°E). After being rinsed with deionised water, it was chilled with liquid nitrogen and stored at –80 °C freezer in SDIP. The total genomic DNA (gDNA) was isolated from 200 mg of young leaves by CTAB protocol (Doyle and Doyle 1987). The concentration and purity of the gDNA was measured by Nanodrop OneC and Agilent 2100 Bioanalyzer. An Illumina DNA library was constructed and sequenced with paired-end reads on Illumina Hiseq 2500 platform. After removing the contaminated and low quality reads, about 7.3 Gb data were mapped to the online published cp genome of *Q. rubra* (GenBank no. JX970937; Alexander and Woeste 2014) using bowtie2 (Langmead and Salzberg 2012). The cp genome annotation was performed using both of DOGMA pipeline (Wyman et al. 2004) and HMMER (Finn et al. 2011), then corrected manually.

The complete cp genome of *Q. coccinea* (GenBank no. MN308055) is a circular DNA molecule with 161,298 bp in size having 36.80% of total GC content. It contains two inverted repeat regions (IRs, 25,850 bp each) with GC content 42.73%, a small single-copy region (SSC, 19,040 bp) with GC content 30.90% and a large single-copy region (LSC, 90,558 bp) with GC content 34.65%. There are 133 genes annotated in the cp genome, containing 88 PCGs, eight rRNA genes and 37 tRNA genes. Eight PCGs, seven tRNA genes and four rRNA genes were duplicated in the IRs. Two PCGs (*ycf3* and *rps12*) harbour two introns each, while eight of them (*rps16*, *rpoC1*, *rpl2*, *rpl16*, *ndhB*, *ndhA*, *clpP* and *atpF*), harbour one intron each.

For phylogenetic maximum likelihood (ML) analysis, multiple alignment was done by MAFFT with 19 published cp genomes downloaded from Genbank (Katoh and Standley 2013). The ML tree, reconstructed using RAxML (Stamatakis 2006), showed that both of *Q. coccinea* and *Q. rubra* were phylogenetically closer to *Q. aliena* var. *acutiserrata* than others (Figure 1).

**Figure 1.**
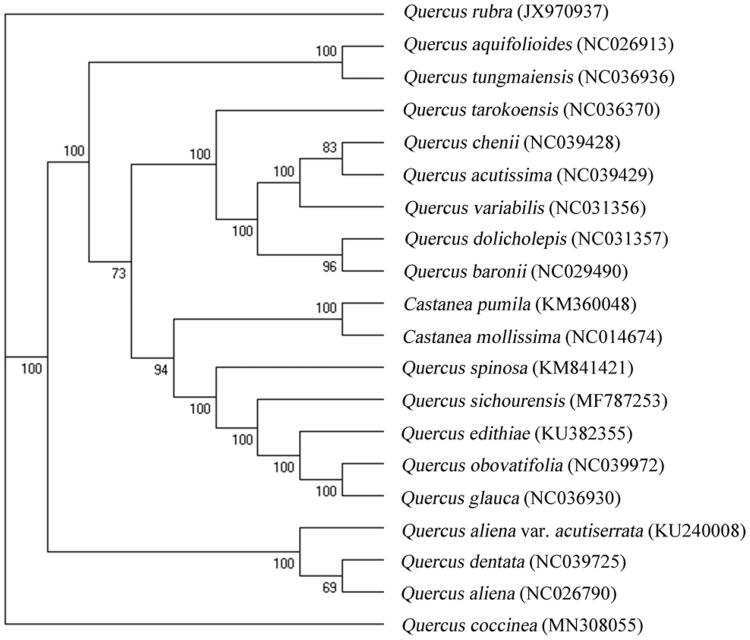
Phylogenetic tree based on 20 complete cp genome sequences. The bootstrap support values are shown next to the branches.
